# Promising antitumor effects of the curcumin analog DMC-BH on colorectal cancer cells

**DOI:** 10.18632/aging.204610

**Published:** 2023-03-24

**Authors:** Gang Liu, Jian Chen, Zhicheng Bao

**Affiliations:** 1 Department of General Surgery, Suzhou Medical College of Soochow University, Suzhou 215300, Jiangsu Province, China; 2 Department of General Surgery, Affiliated Kunshan Hospital of Jiangsu University, Suzhou 215300, Jiangsu Province, China; 3 Department of Rehabilitation, Gusu School, Nanjing Medical University, The First People's Hospital of Kunshan, Suzhou 215300, Jiangsu Province, China

**Keywords:** colorectal cancer, DMC-BH, proliferation, invasion, apoptosis

## Abstract

Colorectal cancer (CRC) is a common malignant tumor of the digestive system worldwide. DMC-BH, a curcumin analog, has been reported to possess anticancer properties against human gliomas. However, its effects and mechanism on CRC cells are still unknown. Our present study demonstrated that DMC-BH had stronger cytostatic ability than curcumin against CRC cells *in vitro* and *in vivo*. It effectively inhibited the proliferation and invasion and promoted the apoptosis of HCT116 and HT-29 cells. RNA-Seq and data analysis indicated that its effects might be mediated by regulation of the PI3K/AKT signaling. Western blotting further confirmed that it dose-dependently suppressed the phosphorylation of PI3K, AKT and mTOR. The Akt pathway activator SC79 reversed the proapoptotic effects of DMC-BH on CRC cells, indicating that its effects are mediated by PI3K/AKT/mTOR signaling. Collectively, the results of the present study suggest that DMC-BH exerts more potent effects than curcumin against CRC by inactivating the PI3K/AKT/mTOR signaling pathway.

## INTRODUCTION

Colon cancer (CRC) is a disease caused by the interaction of genetic and environmental factors and is one of the most common malignant tumors worldwide [1]. Statistics from Siegel et al. show that it is estimated that nearly 140,000 people in the United States will be diagnosed with CRC, and approximately 50,000 people will die from it each year [2]. The incidence is gradually increasing. The above statistics show that colon cancer is a malignant tumor that seriously threatens human life.

At present, the treatment of CRC is mainly surgery-based comprehensive treatment [3]. The resection rate of surgery can reach 60%-70%. After radical resection of CRC, 50% of patients die from recurrence or metastasis, and 55.80% of recurrence events occur within 2 years after surgery. The 5-year survival rate reported in a large number of cases is approximately 50%-60%, and the expansion of the scope of surgery has not improved the survival rate of patients. Chemotherapy can reduce the risk of postoperative recurrence, improve clinical efficacy, reduce the incidence of metastasis, and save patients’ lives or prolong life as much as possible [4]. Although great progress has been made in surgical treatment, recurrence and metastasis after surgery are the main causes of death in CRC patients [5]. Therefore, controlling the recurrence and metastasis of CRC is the key to improving the prognosis and quality of life of patients.

DMC-BH is a compound that was obtained by introducing a water-soluble pyrrolidine fragment into the benzene ring of the natural compound curcumin to improve its water solubility and has antiglioma effects [6, 7]. Curcumin is a well-known plant-derived polyphenol and has been shown to inhibit the progression of certain cancer cells, including CRC [8, 9]. In this study, we found that DMC-BH has stronger anti-CRC effects than curcumin both *in vitro* and *in vivo*. However, its underlying mechanism in CRC remains poorly understood. In the present study, we investigated the potential ability of DMC-BH to suppress CRC and demonstrated the potential mechanism.

## RESULTS

### DMC-BH has stronger cytostatic ability than curcumin

**Figure 1:**
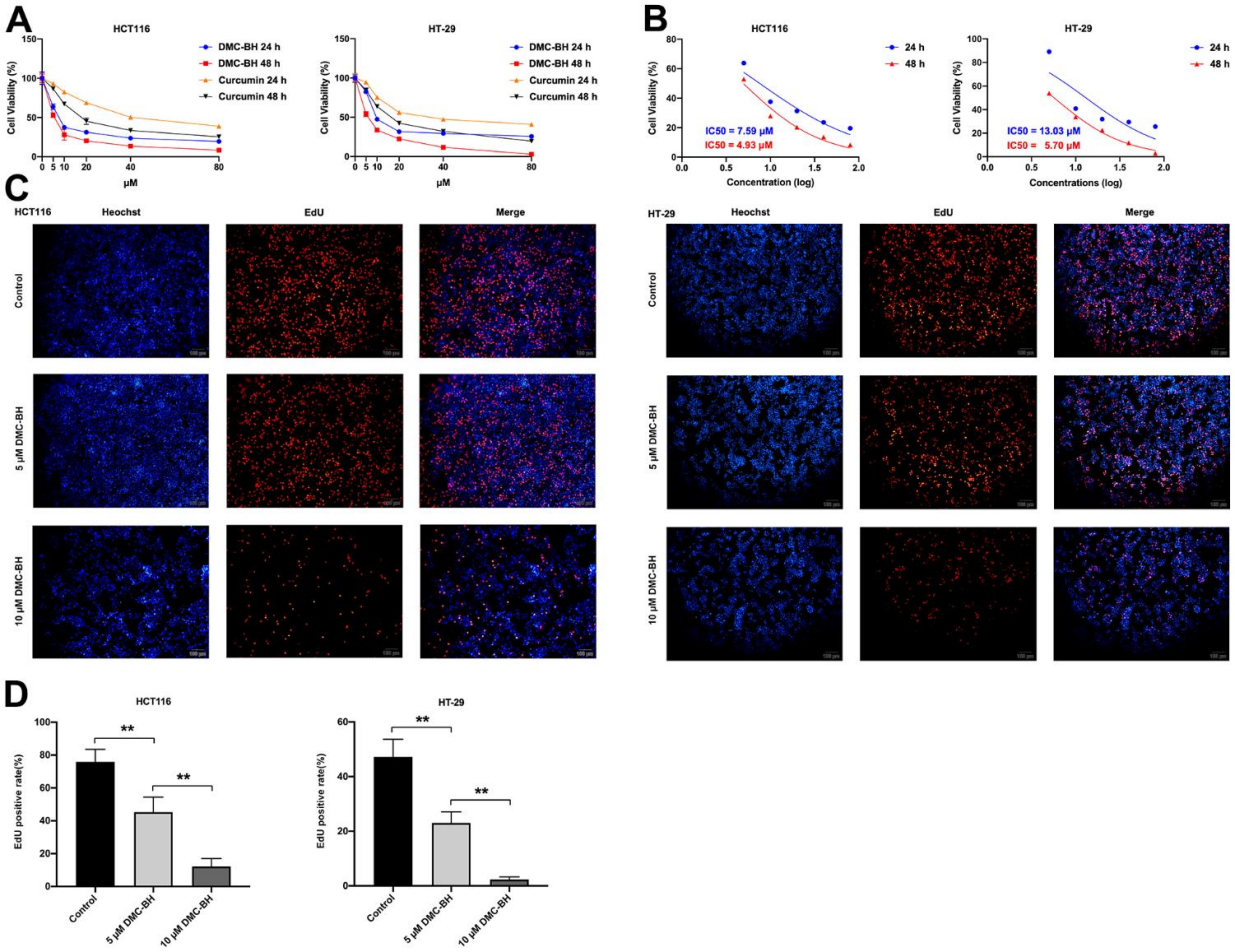
**DMC-BH inhibits the proliferation of HCT116 and HT-29 cells.** (**A**) Effects of DMC-BH and curcumin on the viability of HCT116 and HT-29 cells. (**B**) IC50 values of DMC-BH in HCT116 and HT-29 cells at 24 h and 48 h. (**C**) EdU assay of HCT116 and HT-29 cells after DMC-BH treatment. (**D**) EdU-positive rates in HCT116 and HT-29 cells after DMC-BH treatment.

To evaluate the antiproliferative effects of DMC-BH and its parent compound curcumin on CRC, HCT116 and HT-29 cells were treated with 0, 5, 10, 20, 40, and 80 μM DMC-BH and curcumin for 24 and 48 h and analyzed by the CCK-8 assay method. As shown in Figure 1A, both DMC-BH and curcumin inhibited cell proliferation in a dose- and time-dependent manner; however, DMC-BH exhibited stronger cytostatic ability than curcumin. Based on these data, the IC50 values of DMC-BH in HCT116 and HT-29 cells were calculated to be 7.59 μM and 13.03 μM at 24 h and 4.93 μM and 5.70 μM at 48 h, respectively (Figure 1B).

Furthermore, the EdU method was applied to verify the changes in cell proliferation after DMC-BH treatment. According to the IC50s in HCT116 and HT-29 cells, cells were treated with 5 and 10 μM DMC-BH for 24 h. The EdU assay results showed that the number of EdU-positive CRC cells in the DMC-BH (5 or 10 μM) group was significantly lower than that in the control group (Figure 1C). As shown in Figure 1D, the EdU-positive rate decreased with increasing concentrations of DMC-BH, which indicated that cell proliferation was suppressed by DMC-BH in a dose-dependent manner. These data suggested that DMC-BH had potent antiproliferative effects on CRC cells.

### DMC-BH induced the apoptosis of CRC cells

**Figure 2:**
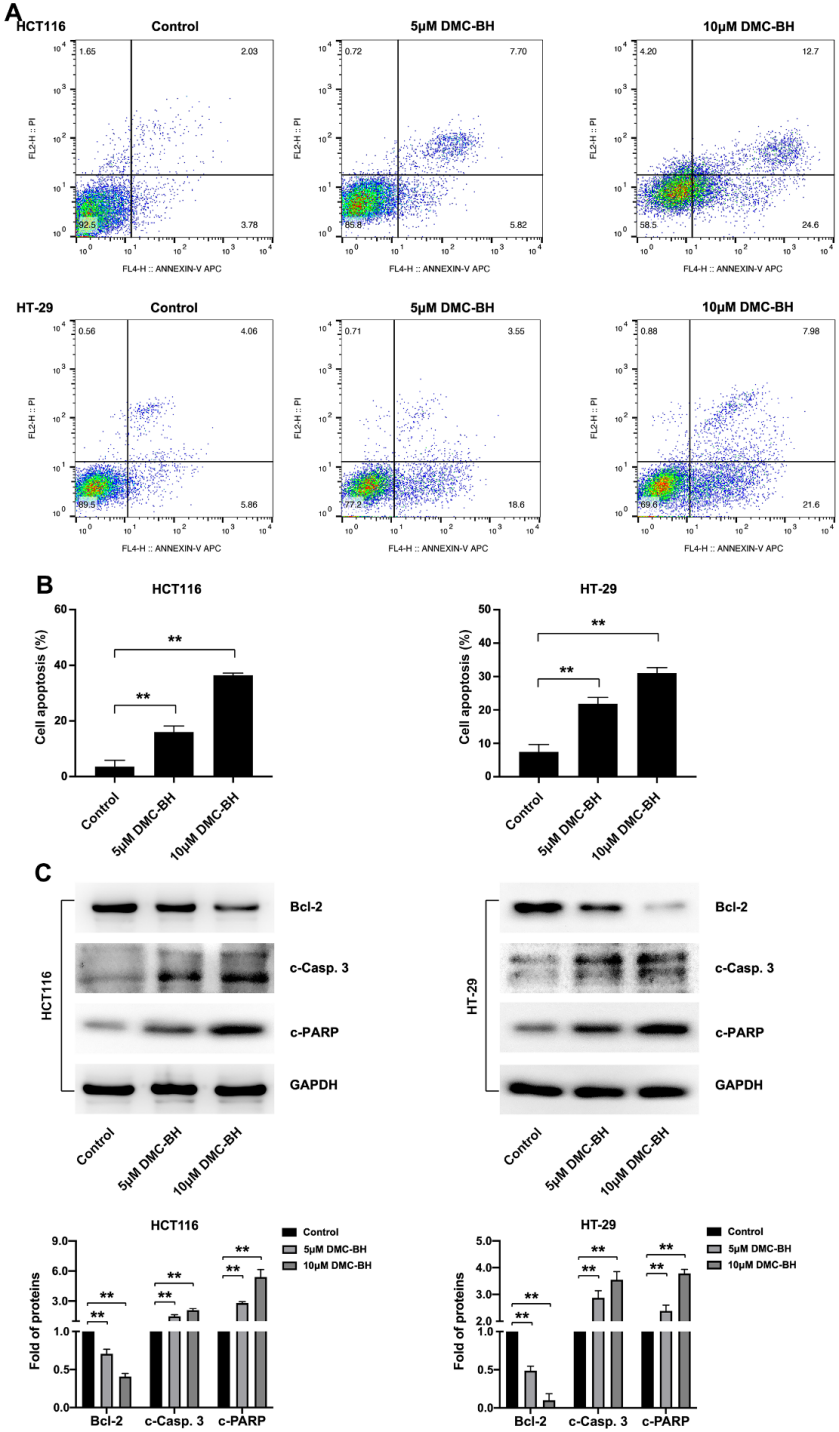
**DMC-BH induces the apoptosis of CRC cells.** (**A**) Flow cytometry assay of HCT116 and HT-29 cells after DMC-BH treatment. (**B**) Apoptosis rates of HCT116 and HT-29 cells after DMC-BH treatment. (**C**) Western blot analysis of the apoptosis-related proteins Bcl-2, c-PARP, and c-Casp. 3.

In the present study, cell apoptosis was further analyzed by Annexin V-APC/PI double staining (Figure 2A). Flow cytometry analysis showed that 5 and 10 μM DMC-BH significantly increased the proportion of apoptotic cells compared to that in the control group (P<0.05), and this effect was dose-dependent (15.98 ± 2.17%, 36.40  ±  0.79% in HCT116 cells; 21.86  ± 1.92%, 31.09 ±  1.60% in HT-29 cells, respectively) (Figure 2B).

Then, the expression of three apoptosis-related proteins, Bcl-2 (an anti-apoptotic protein), caspase-3 (a crucial apoptosis executor) and PARP (a substrate of caspase-3), was further detected in HCT116 and HT-29 cells treated with 5 or 10 μM DMC-BH for 24 h. The results indicated that DMC-BH treatment decreased the expression of Bcl-2 and increased the expression of cleaved caspase-3 (c-Casp. 3) and cleaved PARP (c-PARP) compared with that in the control group (P<0.05) (Figure 2C). These results indicate that DMC-BH induces the apoptosis of CRC cells by inducing the activation of the intrinsic caspase pathway.

### DMC-BH suppressed the migration and invasion of CRC cells *in vitro*

To detect the effects of DMC-BH on the cell migration and metastasis of CRC cells, HCT116 and HT-29 cells were treated with 5 and 10 μM DMC-BH for 12 h and then analyzed by using a Transwell assay with or without Matrigel. In the migration assay, the Transwell chamber was not coated with Matrigel. As shown in Figure 3A, 3C, with increasing DMC-BH concentration, the number of migrating HCT116 or HT-29 cells was significantly reduced on the outer surface and was lower than that in the control group (P<0.05). A similar situation was also observed in invasion experiments containing Matrigel (Figure 3B, 3C). Moreover, the expression of the invasion- and migration-related genes MMP-2 and MMP-9 and the epithelial-to-mesenchymal transition (EMT)-related genes N-cadherin and Snail were further analyzed by Western blot assays. As shown in Figure 3D, MMP-9, MMP-2, N-cadherin and Snail were obviously downregulated in the DMC-BH treatment group and were significantly lower than those in the control group (P<0.05). These data suggest that DMC-BH has obvious inhibitory effects on the migration and metastasis of CRC cells.

**Figure 3:**
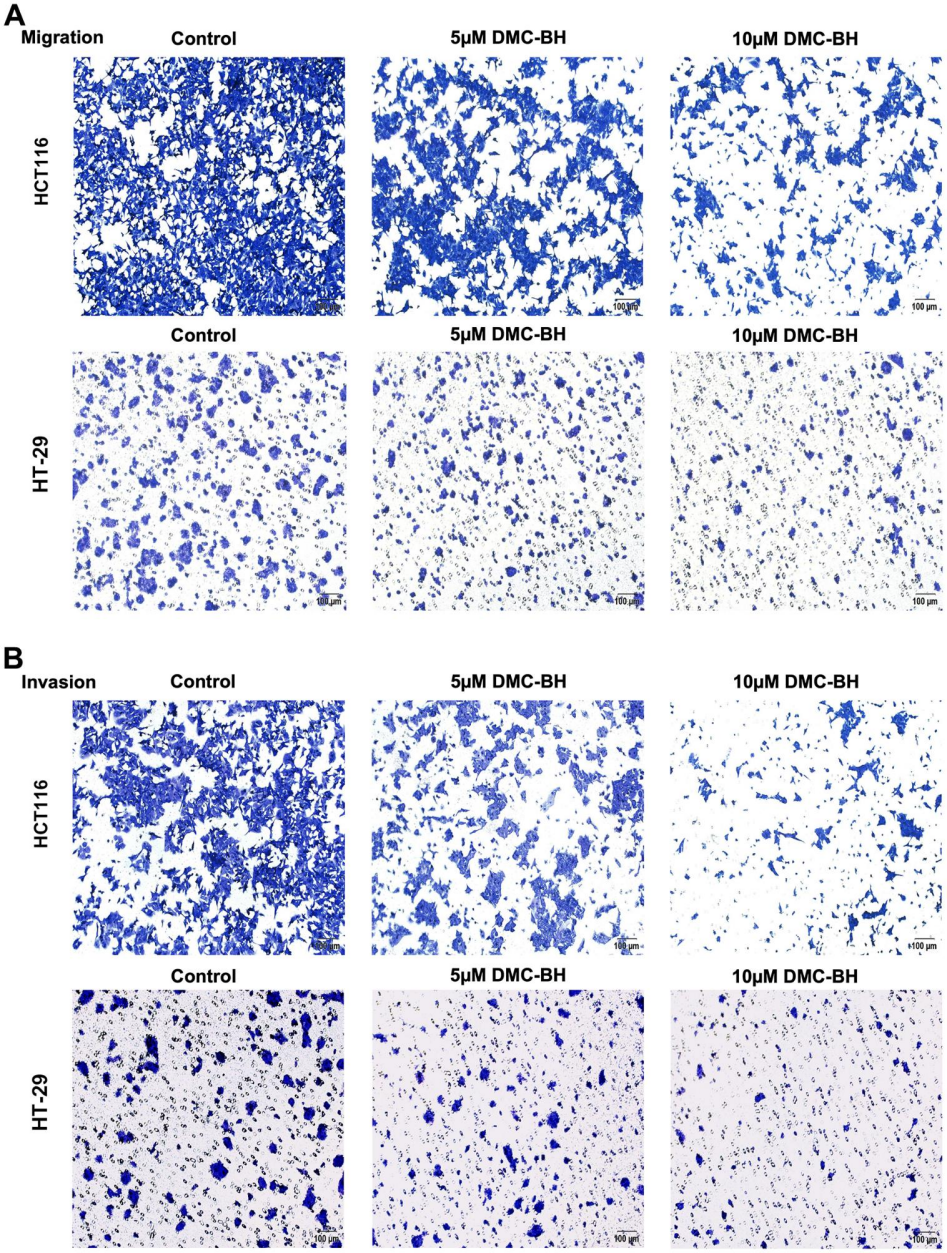
**DMC-BH inhibits the migration and invasion of CRC cells *in vitro*.** (**A**) Migration assay in HCT116 and HT-29 cells after DMC-BH treatment. (**B**) Invasion assay of HCT116 and HT-29 cells after DMC-BH treatment.

**Figure 3:**
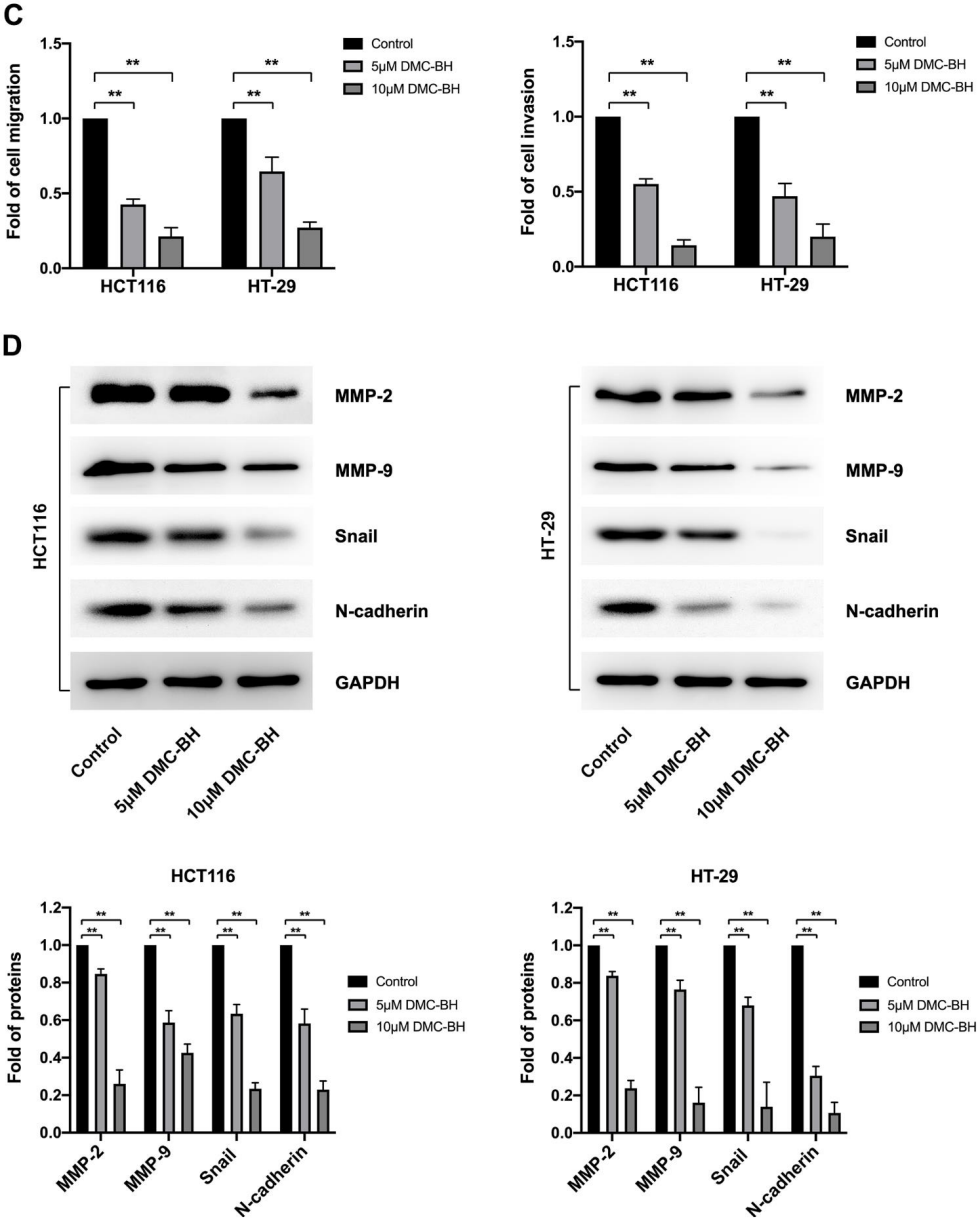
**DMC-BH inhibits the migration and invasion of CRC cells *in vitro*.** (**C**) Statistical analysis of cell migration and invasion in HCT116 and HT-29 cells after DMC-BH treatment. (**D**) Western blot analysis of the changes in MMP9, MMP2, N-cadherin and Snail protein levels.

### Identification and functional enrichment analysis of differentially expressed genes (DEGs)

**Figure 4:**
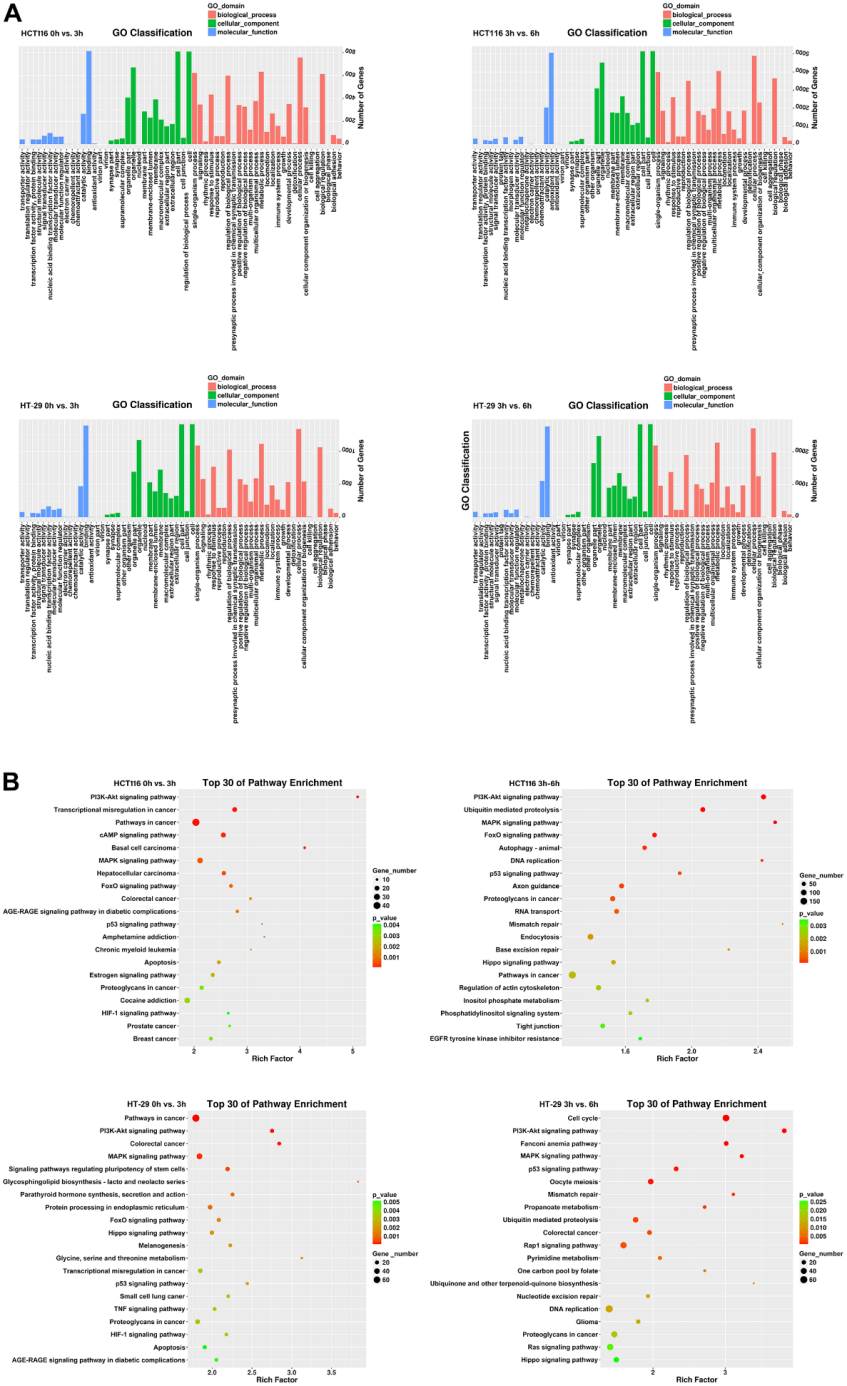
**Identification of DEGs in CRC cells after DMC-BH treatment.** (**A**) GO analysis. (**B**) KEGG analysis.

To investigate the transcriptional changes in HCT116 and HT-29 cells after DMC-BH treatment, the purified mRNA from DMC-BH-treated cells was sequenced on the Proton platform. Based on the RNA-seq data, the data were subjected to Gene Ontology (GO) and Kyoto Encyclopedia of Genes and Genomes (KEGG) analyses. In this study, we compared gene expression between the 0 h- vs. 3 h- and 3 h- vs. 6 h-DMC-BH-treated groups and then identified common changes. As shown in Figure 4A, GO terms showed that 67 genes were enriched in cell growth in the HCT116-3 h group vs the HCT116-0 h group, 329 genes were enriched in cell growth in the HCT116-6 h group vs the HCT116-3 h group, 116 genes were enriched in cell growth in the HT-29-3 h group vs HT-29-0 h group, and 151 genes were enriched in cell growth in the HT-29-6 h group vs the HT-29-3 h group. The KEGG pathway analysis results are shown in Figure 4B. DEGs were mainly enriched in proliferation-related pathways, including the PI3K-Akt signaling pathway and cAMP signaling pathway in the HCT116-0 h group vs. the HCT116-3 h group, while the PI3K-Akt signaling pathway, ubiquitin-mediated proteolysis, MAPK signaling pathway, FoxO signaling pathway, and p53 signaling pathway were enriched in the HCT116-3 h group vs. the HCT116-6 h group. Furthermore, we confirmed the pathway changes in HT-29, and data showed PI3K-Akt signaling pathway and MAPK signaling pathway were enriched in the HT-29-0 h group vs. the HT-29-3 h group, while the cell cycle, PI3K-Akt signaling pathway, MAPK signaling pathway, FoxO signaling pathway, and p53 signaling pathway were enriched in the HT-29-3 h group vs. the HT-29-6 h group. Considering that a larger rich factor means a greater degree of enrichment and consistency of altered signaling pathways in two cell lines, we considered the PI3K-Akt signaling pathway to be a potential related pathway. These results suggest that DMC-BH-induced anti-CRC effects may be associated with the PI3K-Akt signaling pathway.

### The antitumor effects of DMC-BH on CRC cells are mediated by PI3K/Akt/mTOR signaling

**Figure 5:**
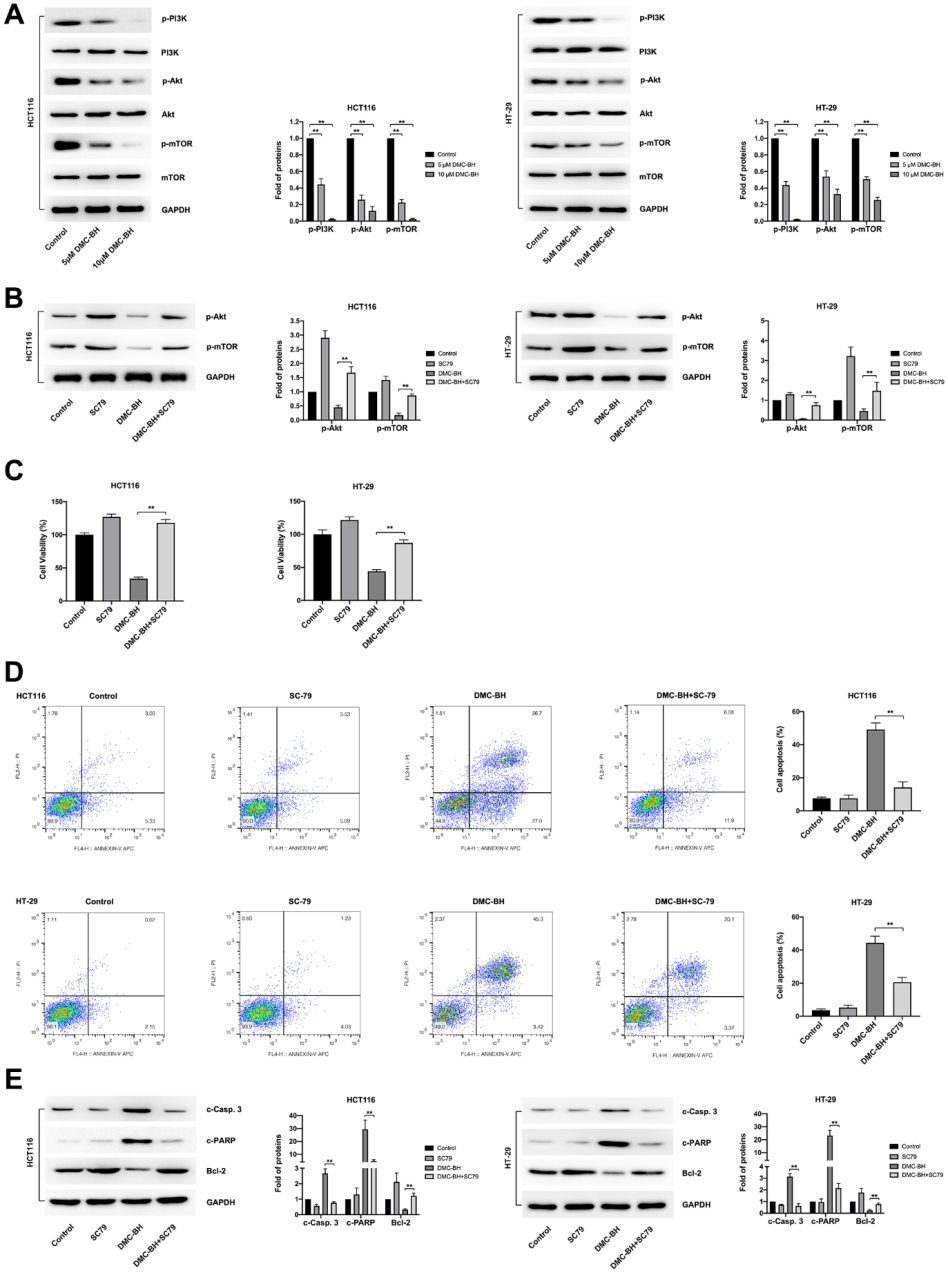
**DMC-BH inhibits PI3K-Akt signaling in CRC cells.** (**A**) Western blot analysis of phosphorylated levels of p-PI3K, p-Akt, and p-mTOR after DMC-BH treatment. (**B**) The phosphorylated levels of p-Akt and p-mTOR measured after incubation with the Akt pathway activator SC79 (10.96 μM). (**C**) Cell viability analysis of CRC cells treated with DMC-BH and SC79 in combination. (**D**) Flow cytometry analysis of CRC cells treated with DMC-BH and SC79 in combination. (**E**) Western blot analysis of the relevant apoptotic proteins (Bcl-2 and c-Casp. 3 and c-PARP) after DMC-BH and SC79 combined treatment.

To further investigate the regulatory pathways of PI3K-Akt signaling based on the data from RNA-seq analysis, we identified the proteins of PI3K-Akt signaling involved in DMC-BH-induced cell proliferation inhibition and apoptosis. To study the effects of DMC-BH on the related protein expression of PI3K-Akt signaling, HCT116 and HT-29 cells were cultured for 24 h and then treated with 5 and 10 μM DMC-BH for 24 h. PI3K-Akt signaling, including PI3K, p-PI3K, Akt, p-Akt, mTOR and p-mTOR, was detected by western blotting. As shown in Figure 5A, compared with the control, DMC-BH significantly reduced the phosphorylation levels of p-PI3K, p-Akt, and p-mTOR. Phosphorylation is the activator of signaling, which means DMC-BH inhibited the activation of the PI3K/Akt/mTOR signaling pathway.

To assess the antitumor effects of DMC-BH, we suppressed the PI3K/Akt/mTOR signaling pathway with the classic Akt pathway activator SC79, which augments Akt phosphorylation at both the Thr308 and S473 sites. As shown in Figure 5B, SC79 (10.96 μM) reversed the effects of DMC-BH (10 μM) on Akt phosphorylation and downstream mTOR phosphorylation. CCK-8 and fluorescence-activated cell sorter (FACS) assays were further applied to confirm the antitumor effects induced by DMC-BH and SC79 in combination. The CCK-8 assay showed that the inhibitory effect on the cell viability of HCT116 and HT-29 cells induced by DMC-BH (10 μM) for 24 h was significantly attenuated by SC79 (Figure 5C). Moreover, flow cytometry assays showed that cells cotreated with DMC-BH (10 μM) and SC79 had a lower apoptosis rate than those treated with DMC-BH alone (14.12 ± 3.37% vs*.* 49.17 ± 3.99% in HCT116 cells; 20.63 ± 2.83% vs*.* 44.36± 4.05% in HT-29 cells, respectively) (Figure 5D). The change in potentially relevant apoptotic proteins also confirmed this. As shown in Figure 5E, cotreatment with DMC-BH (10 μM) and SC79 significantly restored Bcl-2 expression but attenuated the increases in c-Casp. 3 and c-PARP levels that were induced DMC-BH alone, suggesting that the apoptosis process by DMC-BH was suppressed. These data suggest that the Akt activator could reverse the antitumor effects mediated by DMC-BH. Taken together, the above data revealed that the anti-CRC effect of DMC-BH was mediated by PI3K/Akt/mTOR signaling.

### DMC-BH has stronger antitumor effects than curcumin *in vivo*

The *in vivo* antitumor efficacy of DMC-BH and curcumin was analyzed by HCT116 and HT-29 xenograft models. Nude mice were intraperitoneally administered DMC-BH or curcumin daily, and the data showed that either DMC-BH or curcumin had inhibitory effects on xenografts. As shown in Figure 6, the effect of DMC-BH in inhibiting the volume (Figure 6A) and weight (Figure 6B) of the transplanted tumor was significantly higher than that of curcumin (P<0.05). Furthermore, we analyzed the expression of Akt signaling and showed that DMC-BH significantly decreased the expression of p-AKT and p-mTOR by immunohistochemical analysis (Figure 6C) and Western blotting (Figure 6D), indicating that DMC-BH inactivates PI3K/AKT signaling. In addition, DMC-BH inhibited the expression of MMP-2 and MMP-9 (Figure 6E).

**Figure 6:**
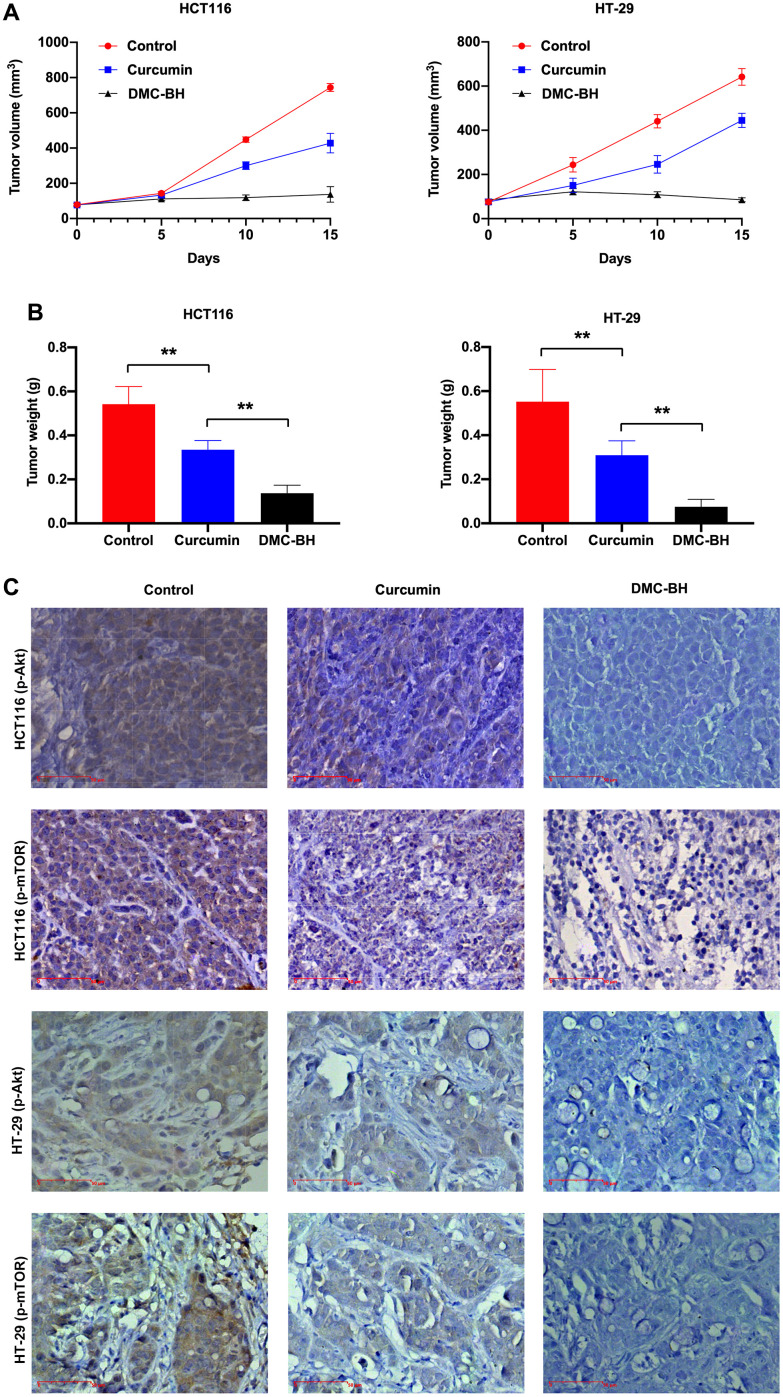
**DMC-BH inhibits the growth of xenografts derived from HCT116 and HT-29 cells.** (**A**) Volumes of xenografts derived from CRC cells treated with 20 mg/kg DMC-BH or curcumin. (**B**) Xenograft weights of CRC cells treated with 20 mg/kg DMC-BH or curcumin. (**C**) Immunohistochemical analysis of p-AKT and p-mTOR expression in xenografts.

**Figure 6:**
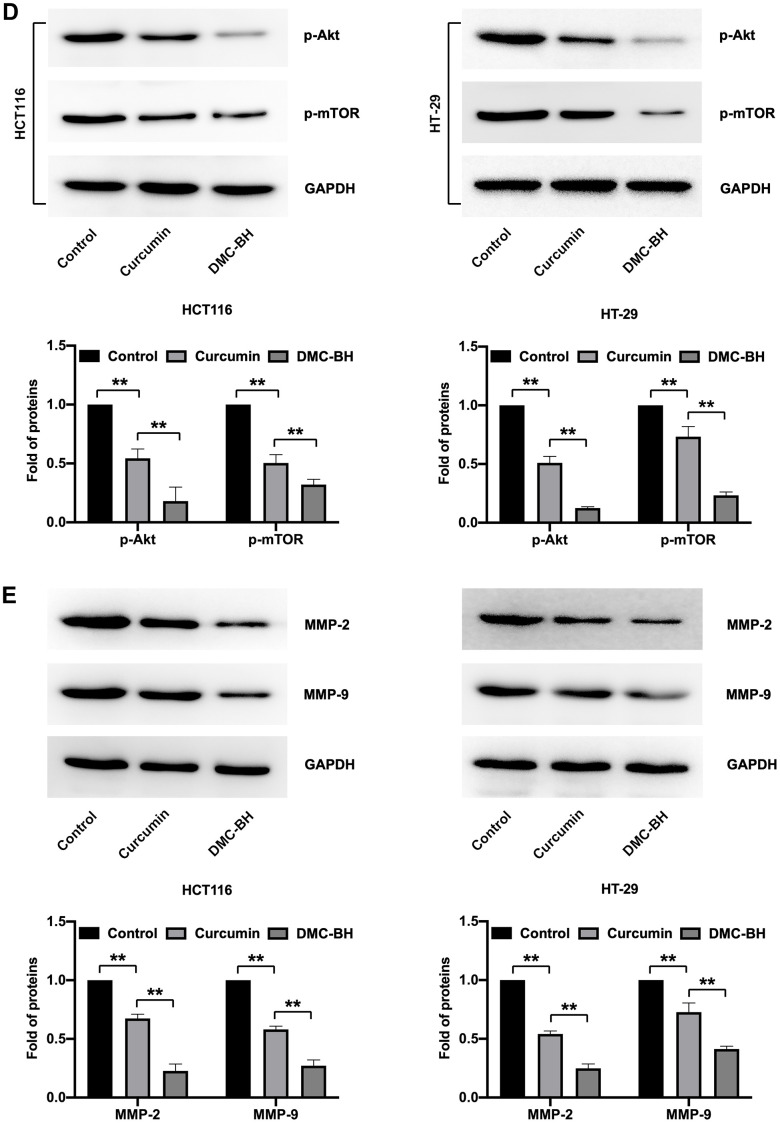
**DMC-BH inhibits the growth of xenografts derived from HCT116 and HT-29 cells.** (**D**) Western blot analysis of p-AKT and p-mTOR expression in xenografts. (**E**) Western blot analysis of MMP-2 and MMP-9 in xenografts.

## DISCUSSION

Currently, the main systemic treatment for CRC is surgery. However, chemotherapy is still indispensable for the treatment of patients after surgery and for the treatment of patients with late-stage disease [10]. Thus, the development of novel chemotherapy drugs has become a matter of great interest. A previous study showed that curcumin had antitumor effects on the cell proliferation, angiogenesis and metastasis of colorectal cancer with an IC50 of 10±0.03 μM for HCT116 cells and 20±0.05 μM for LoVo cells [11]. In the present study, DMC-BH, another analog of curcumin, was confirmed to have stronger anti-CRC effects than curcumin, which has never been reported in previous studies.

In this study, we examined the anti-CRC effects of DMC-BH on HCT116 and HT-29 cells by assessing cell proliferation, migration, invasion and apoptosis *in vitro*. Our data showed that a low dose of 5 μM DMC-BH could effectively inhibit CRC cell proliferation and obviously promote cell apoptosis. Moreover, the cell migration and invasion abilities of CRC cells were abolished to a certain extent. DMC-BH also significantly inhibited the growth of CRC tumors *in vivo* and was more potent than curcumin.

The underlying antitumor mechanisms of DMC-BH in CRC are still unknown. Considering that DMC-BH is a derivative of curcumin, it may affect a signaling pathway similar to that of curcumin in colon cancer. However, Calibasi-Kocal et al. did not explore the mechanism of curcumin in CRC [11]. Mao et al. reported that curcumin decreased the proportion of LGR5(+) CRC stem cells by repressing the oncogenic TFAP2A-mediated extracellular matrix (ECM) pathway [12]. Yu et al. showed that curcumin inhibited the progression of CRC by targeting the lncRNA NBR2/AMPK pathway [13]. Marjaneh et al. inhibited the cell growth and invasive behavior of colitis-associated CRC cells by modulating the Wnt pathway and E-cadherin [14]. The anti-CRC mechanisms of curcumin are inconsistent among studies. Previous research on DMC-BH in gliomas also showed different antitumor mechanisms. It inhibited the renewal of glioma stem cells (GSCs) by activating JNK/ERK signaling, while it suppressed the expansion of glioma cells by inactivating the Atk pathway [6, 7], which indicated that the mechanism of DMC-BH in different cell lines is also inconsistent.

In view of this phenomenon, we performed RNA-seq to explore the DEGs between cells treated or untreated with DMC-BH. According to the RNA-seq data, KEGG pathway enrichment analysis demonstrated that the PI3K-Akt pathway was mainly associated with the anti-CRC effect of DMC-BH. The PI3K/Akt/mTOR signaling pathway plays an important role in the occurrence and development of colon cancer [15]. The PI3K/Akt/mTOR signaling pathway and its downstream signaling molecules are directly involved in regulating the proliferation, apoptosis, invasion and metastasis of colon cancer cells [16–18]. Therefore, this pathway is a hot target pathway for the development of anti-colon cancer drugs. PI3K is a ubiquitous intracellular signal transduction molecule composed of two subunits, p110 and p85, and it can regulate cell proliferation, differentiation and apoptosis [19–21]. Akt, also known as protein kinase B (PKB), is the main downstream effector target of PI3K and is a serine/threonine kinase encoded by the proto-oncogene c-Akt, which results in the occurrence and development of tumors by phosphorylating various cellular transcription factors [22]. It is generally believed that when PI3K is activated by other signals, 3-phosphate phosphatidylinositol (3, 4, 5)-trisphosphate (PIP3) will appear on the plasma membrane and can bind to the PH domain in Akt to make phosphatidyl inositol (3, 4, 5)-trisphosphate [23]. Akt is activated, which in turn activates its downstream important intracellular key molecular target mTOR [24].

In the present study, the main proteins of the PI3K/Akt/mTOR signaling pathway, including PI3K, p-PI3K, Akt, mTOR, phospho (p)-Akt and p-mTOR, were detected, and significantly reduced levels of the p-PI3K, p-Akt, and p-mTOR proteins were confirmed in cells treated with DMC-BH. Phosphorylation is the key process involved in the activation of PI3K/Akt/mTOR signaling. Decreased phosphorylation levels in DMC-BH-treated cells indicated decreased activation of PI3K/Akt/mTOR in CRC cells. Moreover, the Akt pathway activator SC79 reversed the effects of DMC-BH on the phosphorylation of PI3K/Akt/mTOR. CCK-8 and flow cytometry assays also confirmed that SC79 significantly attenuated the proliferation-inhibiting and apoptosis-inducing effects of DMC-BH on CRC cells. These data confirmed the function of DMC-BH in inhibiting the activation of PI3K/Akt/mTOR signaling in CRC.

The regulation of apoptosis by mTOR is mainly related to its different downstream target proteins, including Bcl-2 [25]. Jiang et al. confirmed that mTOR can promote the upregulation of Bcl-2 and enhance cell survival; in contrast, it inhibits Bcl-2 expression, reduces the cell survival rate, and promotes cell apoptosis in CRC [26]. Bcl-2 is an important effector molecule downstream of the PI3K/AKT signaling pathway and is closely related to the negative regulation of apoptosis [27]. When PI3K-dependent AKT is activated, Bcl-2 is depolymerized from phosphorylated Bad, and free Bcl-2 is anchored to the mitochondrial outer membrane, preventing the release of cytochrome c from mitochondria and the activation of caspase-3, exerting anti-apoptotic effects [28]. Activated Akt can regulate the expression of apoptosis-suppressing genes Bcl-2, caspase-3, caspase-9 and other proteins to mediate apoptosis in colon cancer [29]. In this study, the Western blotting results showed that DMC-BH effectively reduced the expression of Bcl-2 in a concentration-dependent manner and increased c-Casp. 3 and c-PARP expression in CRC cells. Decreased expression of Bcl-2 and increased expression of c-Casp. 3 and c-PARP are usually associated with cell apoptosis, which was confirmed by Annexin V-APC/PI double staining analysis in DMC-BH-treated CRC cells. Furthermore, SC79 reversed the DMC-BH-induced changes in Bcl-2, c-Casp. 3 and c-PARP expression in CRC cells.

In conclusion, our study demonstrates that DMC-BH effectively inhibits the proliferation, migration, and invasion and induces the apoptosis of CRC cells by activating the PI3K/AKT/mTOR signaling pathway. These findings suggest that DMC-BH may be a potential therapeutic agent for the treatment of CRC.

## MATERIALS AND METHODS

### Reagents

DMC-BH was donated by Professor Shi's team. The CCK-8 assay kit, Annexin V-FITC/PI Apoptosis Detection Kit and Akt agonist SC79 were purchased from Suzhou Genbio Biotechnology Co., Ltd. (Suzhou, China). An EdU cell proliferation detection kit was purchased from KeyGEN BioTECH (Nanjing, China). The Apoptosis-Hoechst Staining Kit was purchased from Beyotime Biotechnology Co., Ltd. (Shanghai, China). Primary antibodies against PI3K, p-PI3K, AKT, p-AKT, mTOR, p-mTOR, caspase-3, cleaved caspase-3, Bcl-2, Bax and GAPDH were obtained from Abcam (Cambridge, UK). Fetal bovine serum was obtained from Gibco (Grand Island, NY, USA).

### Cell line and culture conditions

The human colon cancer cell lines HT-29 and HCT116 were purchased from Shanghai Chinese Academy of Sciences Cell Bank. HT-29 and HCT116 cells were cultured in Dulbecco's modified Eagle's medium (DMEM) supplemented with 10% fetal bovine serum (FBS) at 37° C in a humidified atmosphere with 5% CO_2_. The medium was changed and the cells were passaged every two days when they were approximately 90% confluent. DMC-BH was dissolved in dimethyl sulfoxide (DMSO) to a stock concentration of 10 mM. Culture media with 0.1% DMSO was used as a control.

### CCK-8 assay for cell proliferation

The cell proliferation assay was detected by CCK-8 assay. A total of 5 × 10^3^ HT-29 and HCT116 cells were seeded into 96-well plates and cultured in DMEM with 10% FBS at 37° C and 5% CO_2_. After overnight incubation, cells were treated with 0, 5, 10, 20, 40 and 80 μM DMC-BH for 48 h. Then, a CCK-8 solution was added to each well according to the manufacturer's instructions at the indicated time. The absorbance of optical density (OD) was measured at 450 nm by using an ELISA plate reader. Untreated cells served as a negative control, while wells without added cells served as blank controls. Based on the data from the CCK-8 assay, the half maximal inhibitory concentration (IC50) of DMC-BH in HT-29 and HCT116 cells was calculated.

### Annexin V/FITC apoptosis assay

HT-29 and HCT116 cells (2×10^5^ cells per well) were seeded into 6-well dishes and incubated with 5 and 10 μM DMC-BH for 24 and 48 h. Then, the cells were harvested, washed with ice-cold phosphate-buffered saline (PBS) and suspended in 1X Annexin V-binding buffer. After that, the cells were stained with 5 μL fluorescein isothiocyanate-conjugated Annexin V-FITC and 10 μL PI for 15 min at room temperature. After staining, the cells were finally analyzed by using a flow cytometer (BD Biosciences, Franklin Lakes, NJ, USA).

### Transwell assays

BD Transwell chambers were coated with Matrigel for the Transwell invasion assay and without Matrigel for the migration assay (BD Biosciences, Franklin Lakes, NJ, USA). HT-29 and HCT116 cells were treated with 5 and 10 μM DMC-BH for 12 h. Then, a total of 1×10^5^ cells per chamber were added to the top chamber with serum-free DMEM, while DMEM with 10% FBS was added to the bottom chamber at 37° C and 5% CO_2_. After 24 h of incubation, the cells on the inner surface of the upper chamber were removed. Then, the invaded cells on the outer surface were fixed with 4% paraformaldehyde (KeyGEN BioTECH, Nanjing, China) and stained with 0.1% crystal violet solution (Sigma-Aldrich, St. Louis, MO, USA) for 5 min. The invading cells were counted in three independent fields (100×) under a microscope.

### Western blotting for protein detection

Following the manufacturer's requirements (Bio-Rad Laboratories, Inc., Hercules, CA, USA), the protein was extracted with RIPA lysis buffer (Genbio, Suzhou, China) and quantified by a NanoDrop 2000 (Thermo Fisher Scientific, Waltham, MA, USA). After adding loading buffer (Genbio, Suzhou, China), equal amounts of protein extracts were added into the lane and separated by SDS-PAGE under a condition of 90 V constant voltage and then transferred to polyvinylidene difluoride membranes (PVDF) using 300 mA current. After blocking with 10% nonfat dry milk, the membranes were incubated with primary antibodies at 4° C overnight. Then, the membranes were washed with T-BST three times. The membranes were incubated with horseradish peroxidase (HRP)-conjugated anti-rabbit/mouse IgG for 1.5 h. The signal was detected with SuperSignal West Pico PLUS (Thermo Fisher Scientific, Waltham, MA, USA).

### Caspase-3 activity assays

After HT-29 and HCT116 cells were treated with 5 and 10 μM DMC-BH for 12 h, cells were collected and washed with PBS twice. According to the manufacturer's requirements (Beyotime Biotechnology, Shanghai, China), 5×10^6^ cells were lysed and clarified by centrifugation at 12,000 × g for 10 min at 4° C. Ten microliters of the lysis supernatant containing approximately 50 μg protein was added to 90 μl of detection buffer. Then, 10 μl of Ac-DEVD-pNA was added and reacted at 37° C for 1-2 hours in the dark, and detection was carried out when a yellow color was produced. The activity of caspase-3 was measured in cell lysates at 405 nm using a microplate reader (M200; TECAN, Japan).

### Cell cycle assays

HT-29 and HCT116 cells (5×10^5^ cells per well) were seeded into 6-well dishes and incubated with 5 and 10 μM DMC-BH for 24 h. The cells were harvested and fixed in 1 ml of ice-bath precooled 70% ethanol and kept at 4° C overnight. The cells were then centrifuged at 1000 × g for 3-5 min. Each tube of cell samples was added to 0.5 ml of propidium iodide staining solution and incubated at 37° C for 30 min in the dark. Then, the cell cycle was analyzed by a flow cytometer (BD Biosciences, Franklin Lakes, NJ, USA).

### EdU incorporation assay

A total of 1×105 HT-29 and HCT116 cells per well were seeded into 96-well dishes and incubated with 5 and 10 μM DMC-BH for 24 h. Then, an equal volume of 2X EdU working solution (20 μM) prewarmed at 37° C was added to each well, and the cells were incubated for an additional 2 h. After EdU labeling of cells was completed, the culture medium was removed, and 1 ml of 4% paraformaldehyde was added. Remove the fixative and wash the cells 3 times with 1 ml of washing solution per well for 3-5 min each. The wash solution was removed, and each well was incubated with 1 ml of PBS containing 0.3% Triton X-100 for 10-15 min at room temperature. The permeabilizer was removed, and the cells were washed 1-2 times with 1 ml of washing solution per well for 3-5 min each time. Then, 0.5 ml of Click reaction solution was added to each well and incubated at room temperature for 30 minutes in the dark. Then, the click reaction solution was removed by suction and washed with washing solution 3 times for 3-5 min each time. After removing the washing solution, 1 ml of 1X Hoechst 33342 solution was added to each well and incubated at room temperature for 10 minutes in the dark. The 1X Hoechst 33342 solution was aspirated, and then the cells were washed 3 times with washing solution for 3-5 min each. The stained cells were observed using a fluorescence microscope (Olympus, Tokyo, Japan).

### RNA-Seq and data analysis

HT-29 and HCT116 cells were treated with 10 μM DMC-BH for 3 and 6 h. Then, total RNA was extracted by TRIzol (Thermo Fisher Scientific, Waltham, MA, USA). The mRNA was further purified by a RNeasy mini kit (Qiagen, Germany). The libraries were generated and subsequently sequenced by an Illumina HiSeq X-ten (SHBIO, Shanghai, China). We used edgeR to analyze differentially expressed genes between samples, performed multiple hypothesis test correction after obtaining the p value, and determined the threshold of the p value by controlling the false discovery rate (FDR). The differential gene screening conditions were as follows: 1) q-value <= 0.05 and 2) fold-change>=2.

### Xenograft model

The *in vivo* antitumor effects of DMC-BH were determined by performing an HCT116 and HT-29 xenograft mouse model. Six-week-old male nude mice were subcutaneously inoculated with 5 × 10^6^ HCT116 or HT-29 cells in the left flank. When the xenograft volume reached 75 mm^3^, DMC-BH or curcumin (20 mg/kg) was administered intraperitoneally once a day for 15 days [6, 30, 31]. Tumor volume was measured every other day with a caliper and calculated using the following formula: V (mm^3^) = Length ×Width^2^ × 0.5. At the end of the experiment, mice were sacrificed, and xenografts were collected for preservation in liquid nitrogen or embedded in paraffin. All animal experimental protocols were approved by the animal ethical protection committee of the First People’s Hospital of Kunshan.

### Statistical analysis

All tests were performed using SPSS Graduate Pack 19.0 statistical software (SPSS, Chicago, IL). Descriptive statistics, including the mean and SE, and one-way ANOVA were used to determine significant differences. *, P < 0.05 and **, P <0.01 were considered statistically significant.
